# Supplementary material for: U-Net Architecture for Prostate Segmentation: The Impact of Loss Function on System Performance
Source: Bioengineering (Basel). 2023 Mar 26;10(4):412. doi: 10.3390/bioengineering10040412 (PMC10135670; doi:10.3390/bioengineering10040412)
Supplement: Supplementary file 1 [file bioengineering-10-00412-s001.zip › bioengineering-2233845-supplementary.pdf]

Supplementary material

Figure S1: Box plots of all metrics used in this study for the *prostate mid-gland* on validation data from the five-fold cross-validation for models with different loss functions.

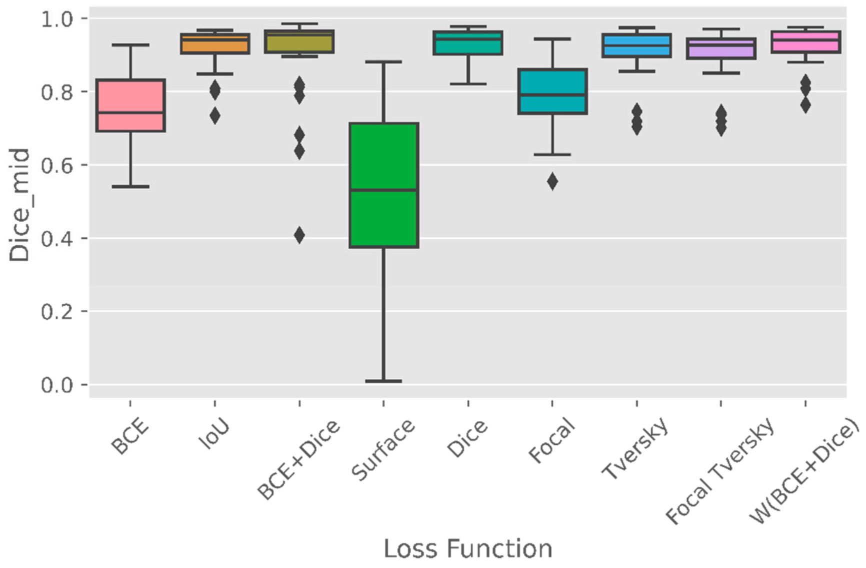

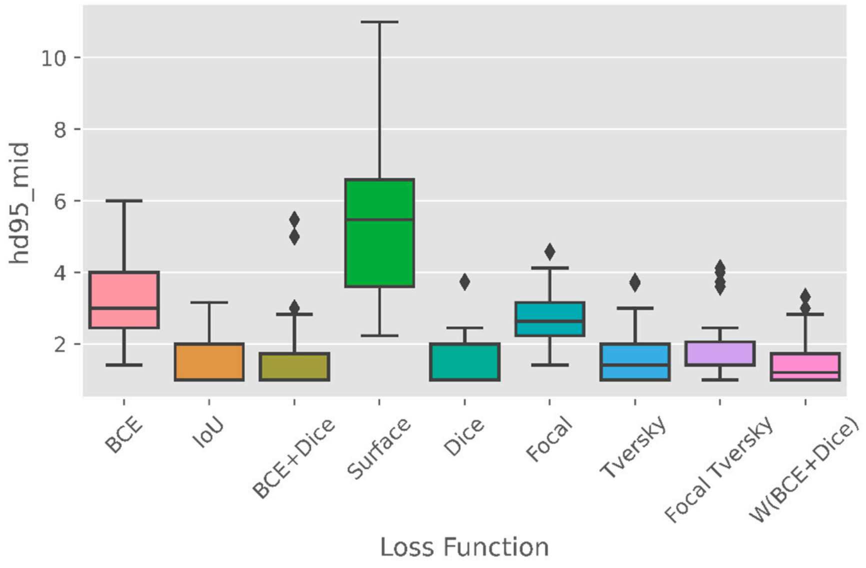

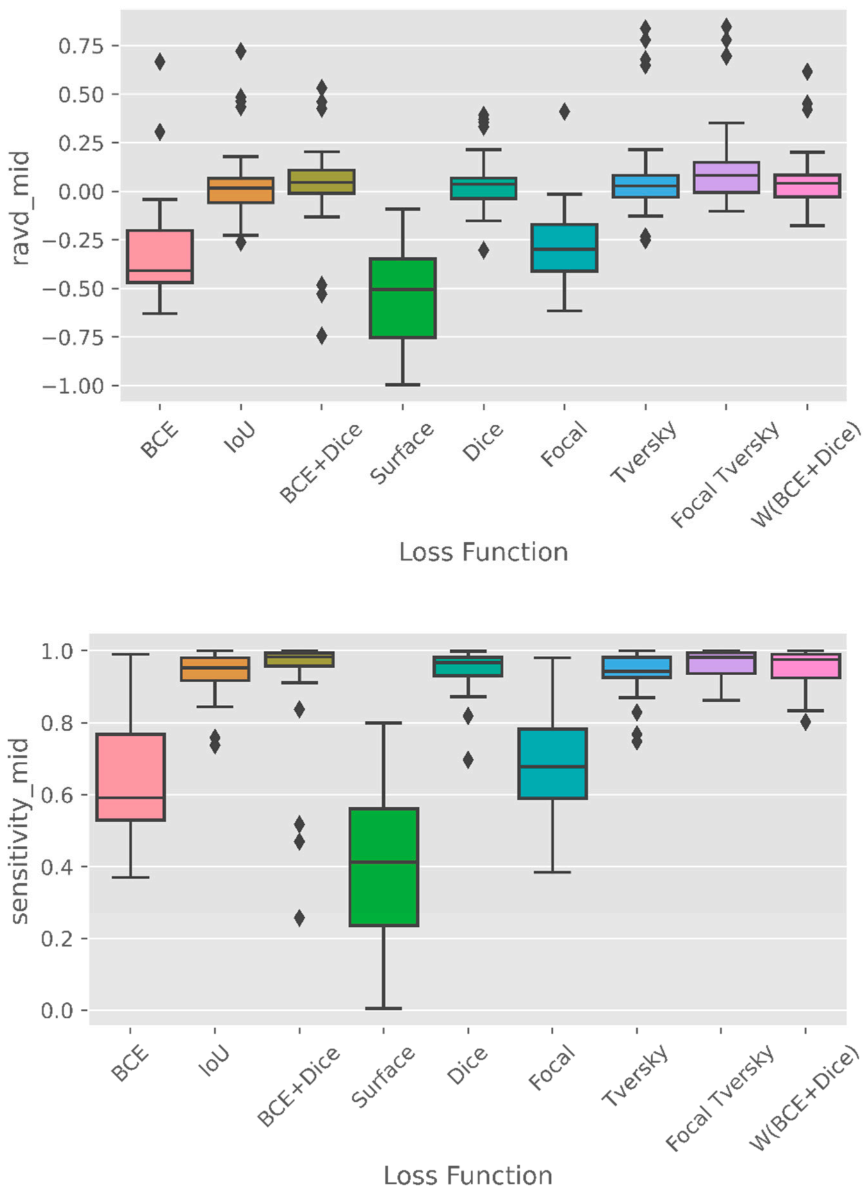

Figure S2: Box plots of all metrics used in this study for the *apex region* on validation data from the five-fold cross-validation for models with different loss functions.

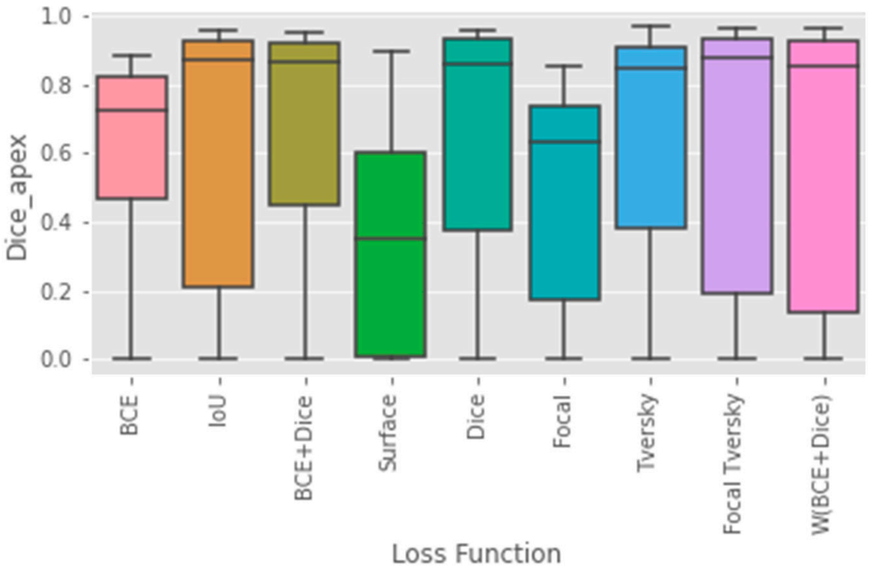

1.0
0.8
0.6
0.4
0.2
0.0
Dice_apex
BCE
IoU
BCE+Dice
Surface
Dice
Focal
Tversky
Focal Tversky
W(BCE+Dice)
Loss Function

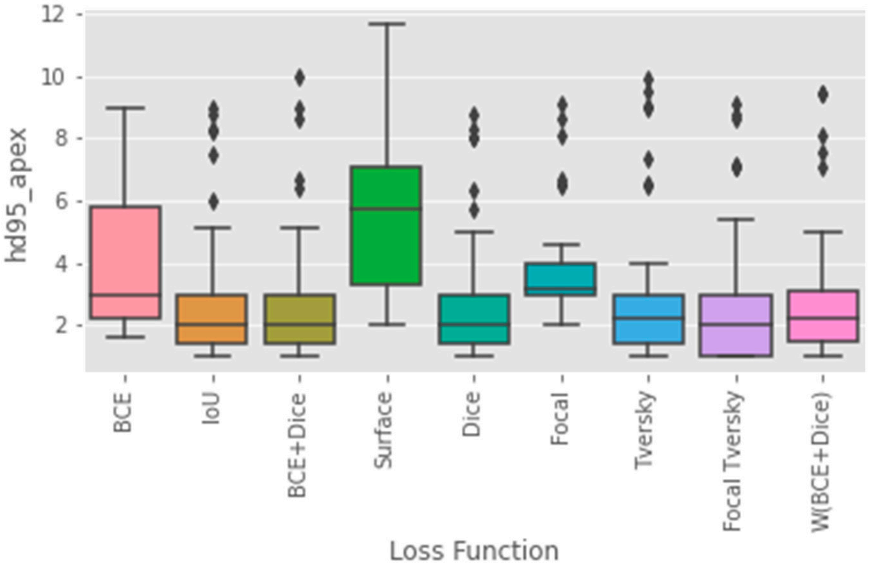

12
10
8
6
4
2
hd95_apex
BCE
IoU
BCE+Dice
Surface
Dice
Focal
Tversky
Focal Tversky
W(BCE+Dice)
Loss Function

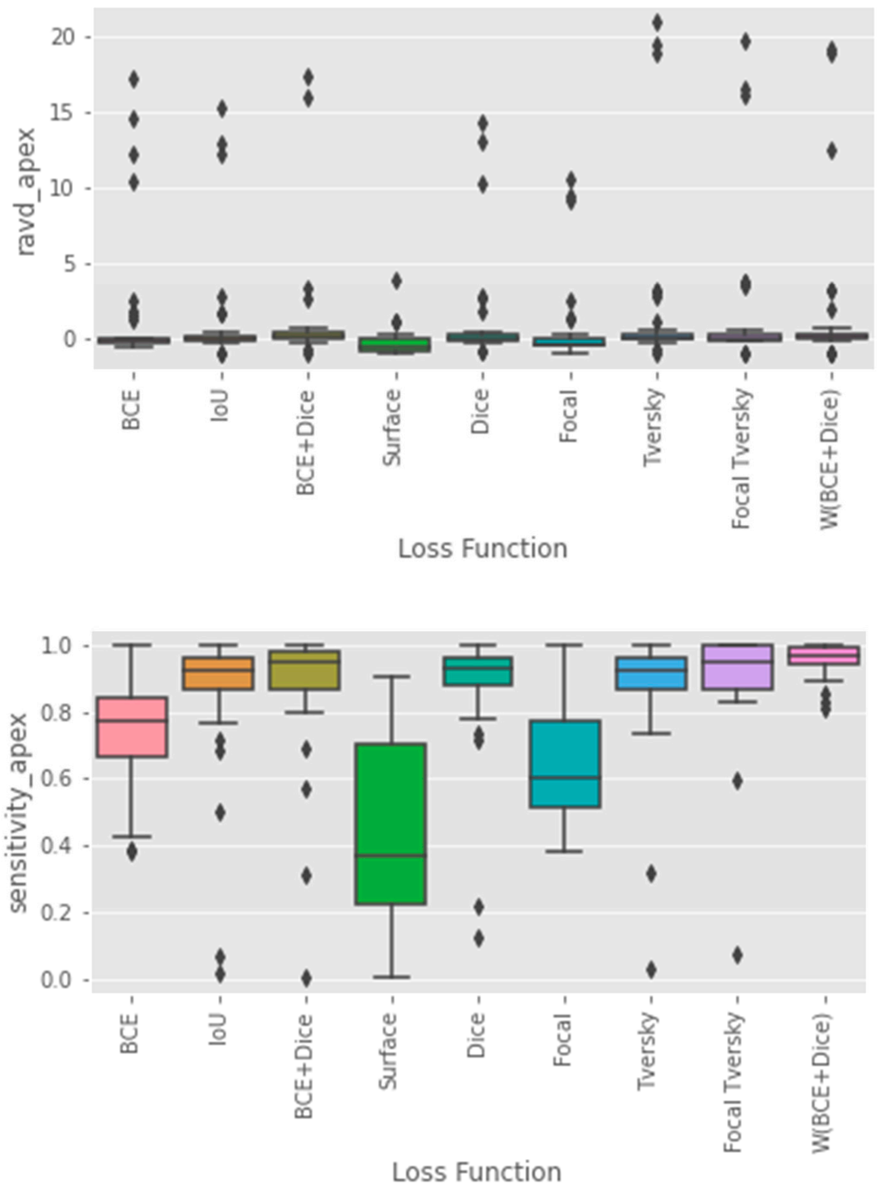

Figure S3: Box plots of all metrics used in this study for the prostate *base region* on validation data from the five-fold cross-validation for models with different loss functions.

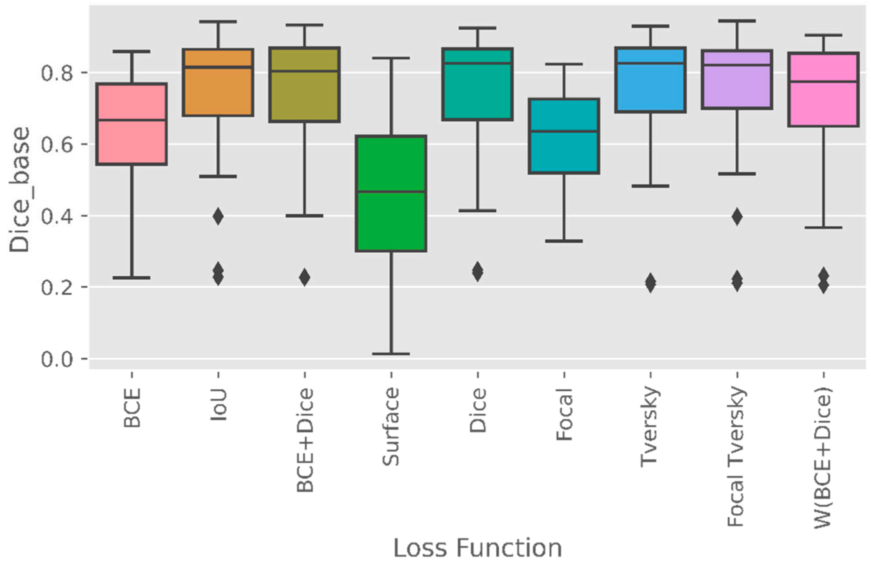
Dice_base
0.0
0.2
0.4
0.6
0.8
BCE
IoU
BCE+Dice
Surface
Dice
Focal
Tversky
Focal Tversky
W(BCE+Dice)
Loss Function

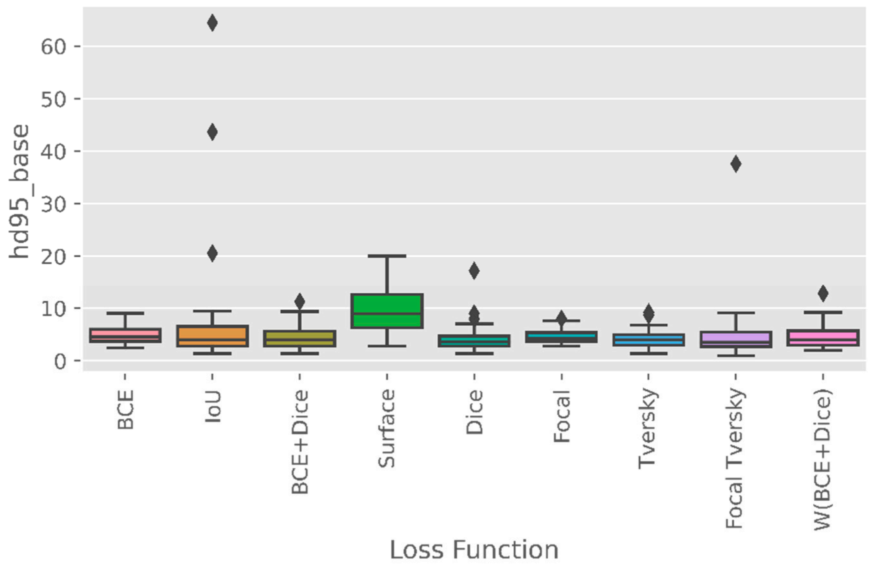
hd95_base
0
10
20
30
40
50
60
BCE
IoU
BCE+Dice
Surface
Dice
Focal
Tversky
Focal Tversky
W(BCE+Dice)
Loss Function

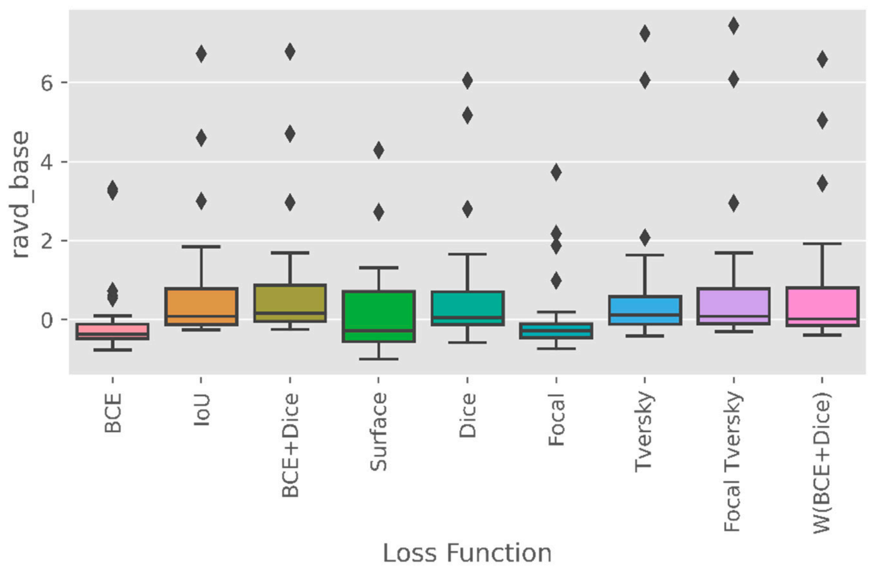

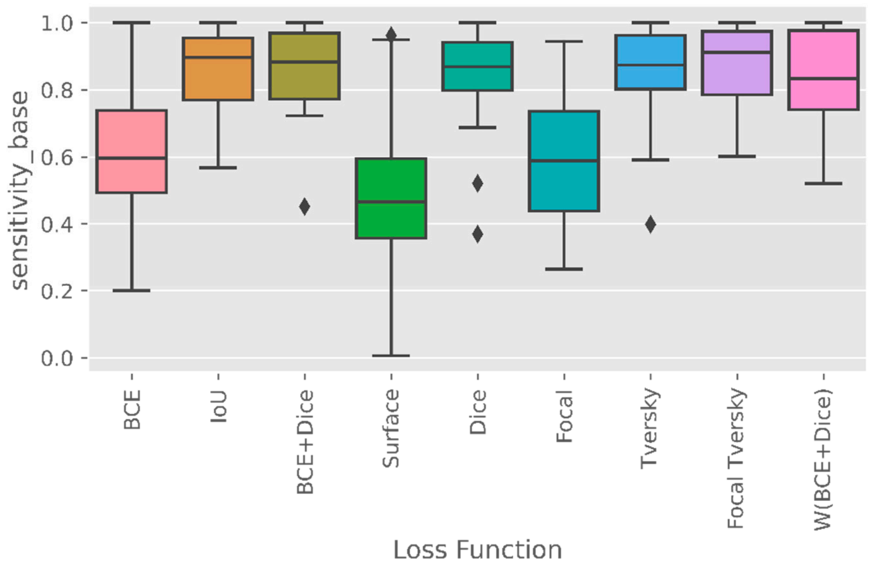

Figure S4: Dice similarity coefficient (DSC) score of all the models for each patient .

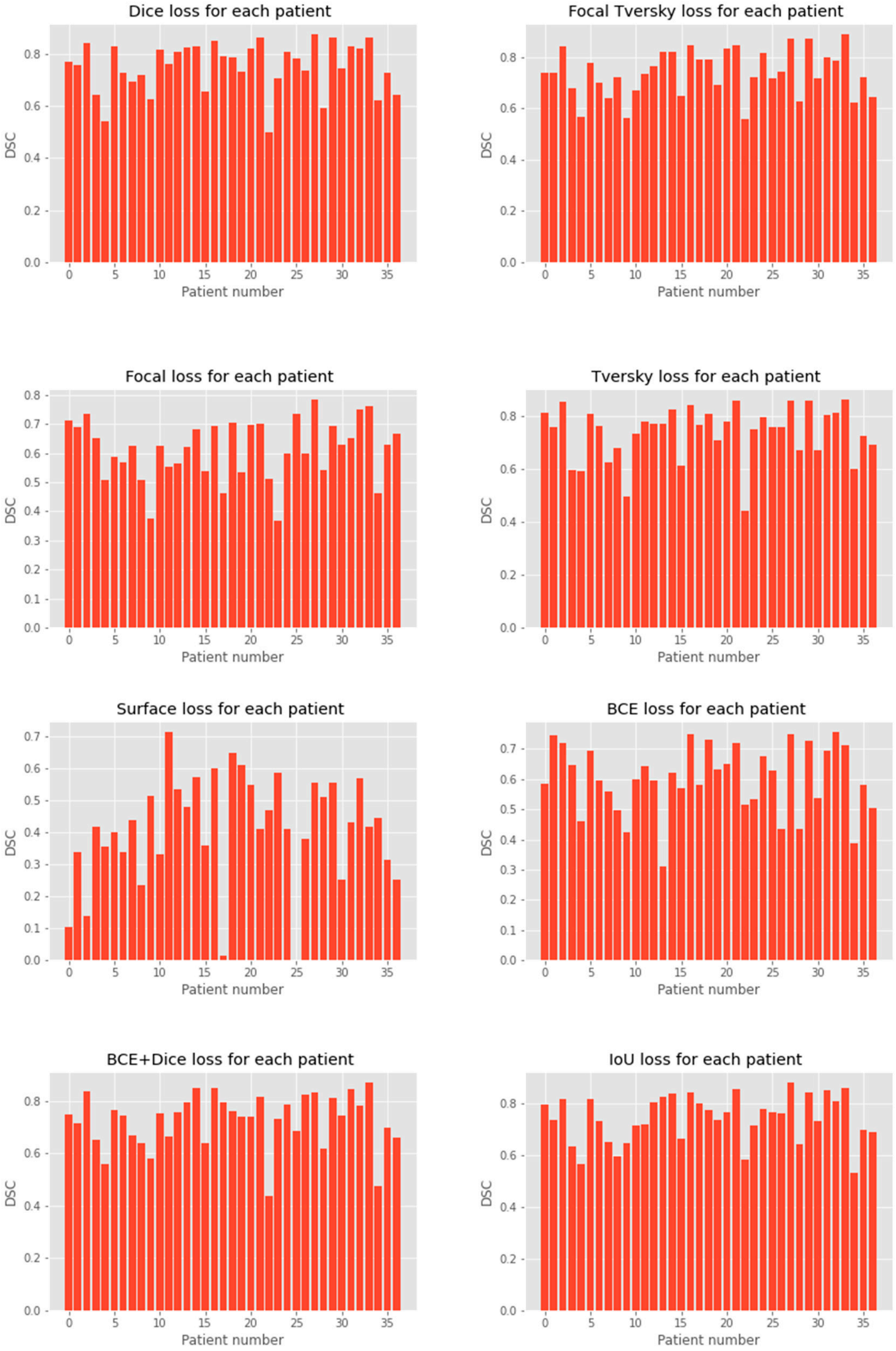

Dice loss for each patient
Focal Tversky loss for each patient
Focal loss for each patient
Tversky loss for each patient
Surface loss for each patient
BCE loss for each patient
BCE+Dice loss for each patient
IoU loss for each patient
DSC
Patient number
0.0
0.1
0.2
0.3
0.4
0.5
0.6
0.7
0.8
0
5
10
15
20
25
30
35

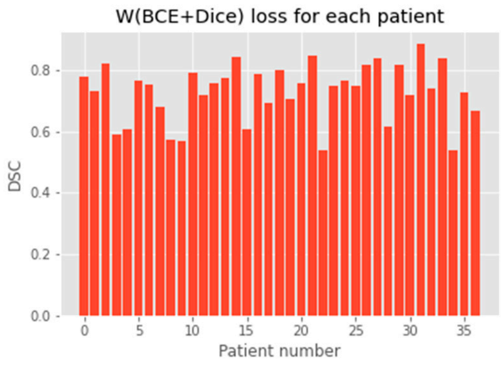

Figure S5: DSC score vs prostate volume (mm^3) for model using Focal Tversky loss. **The limited number of samples prohibits clear conclusions, however it would appear a lower DSC score is apparent compared with larger volumes.**

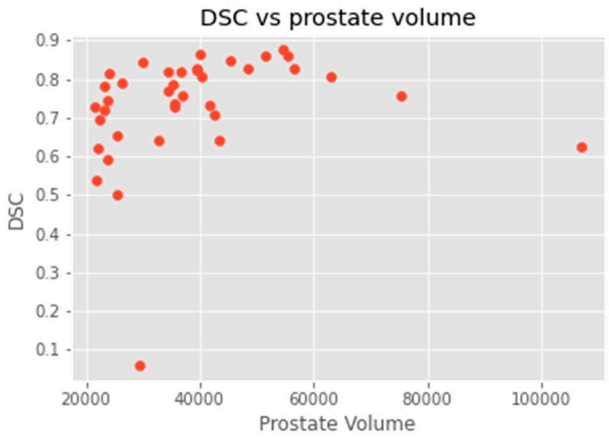

Table S1- W(BCE+Dice), Tversky and Focal Tversky performances, DSC score, for each patient.

Patient No	W(BCE+Dice)	Focal Tversky	Tversky
0	0.78	0.74	**0.81**
1	0.73	0.74	0.76
2	**0.82**	**0.84**	**0.85**
3	0.69	0.68	0.59
4	0.61	0.57	0.59
5	0.76	0.78	**0.81**
6	0.75	0.70	0.76

7	0.68	0.64	0.62
8	0.57	0.72	0.68
9	0.57	0.57	0.50
10	0.79	0.67	0.73
11	0.72	0.73	0.78
12	0.76	0.76	0.77
13	**0.77**	**0.82**	**0.77**
14	**0.84**	**0.82**	**0.82**
15	0.61	0.65	0.61
16	**0.79**	**0.85**	**0.84**
17	0.69	0.79	0.76
18	**0.80**	**0.79**	**0.81**
19	0.70	0.69	0.71
20	0.75	0.84	0.78
21	**0.85**	**0.85**	**0.86**
22	0.54	0.56	0.44
23	0.75	0.72	0.75
24	0.76	0.81	0.80
25	0.75	0.72	0.76
26	**0.82**	**0.74**	**0.76**
27	**0.84**	**0.87**	**0.86**
28	0.62	0.63	0.67
29	**0.82**	**0.87**	**0.86**
30	0.72	0.72	0.67
31	**0.76**	**0.80**	**0.80**
32	0.76	0.79	0.81
33	**0.83**	**0.87**	**0.86**
34	0.76	0.62	0.60
35	0.76	0.72	0.72
36	0.77	0.64	0.69
